# Genomic and Virulence Characteristics of *Brucella intermedia* Isolated from Hospital Wastewater in Ghana

**DOI:** 10.3390/pathogens14060522

**Published:** 2025-05-23

**Authors:** Runa Furuya, Satomi Takei, Yoko Tabe, Anthony Ablordey, Ryoichi Saito

**Affiliations:** 1 Department of Molecular Microbiology and Immunology, Graduate School of Medicine and Dental Science, Institute of Science Tokyo, 1-5-45 Yushima, Bunkyo-ku, Tokyo 113-8510, Japan; furuya.runa@tmd.ac.jp; 2 Department of Clinical Laboratory Medicine, Juntendo University Graduate School of Medicine, 2-1-1 Hongo, Bunkyo-ku, Tokyo 113-8421, Japan; stakei@juntendo.ac.jp (S.T.), tabe@juntendo.ac.jp (Y.T.); 3 Department of Bacteriology, Noguchi Memorial Institute for Medical Research, University of Ghana, Legon P. O. Box LG 25, Ghana; aablordey@noguchi.ug.edu.gh

**Keywords:** antimicrobial resistance, *Brucella intermedia*, cyclic ß-1,2 glucan synthase, hospital wastewater, whole-genome sequencing

## Abstract

*Brucella intermedia*, a gram-negative, non-lactose-fermenting, aerobic, rod-shaped bacterium, is found in environmental sources (e.g., soil and water). In 2020, *Ochrobactrum* was reclassified as *Brucella*. We conducted a genomic analysis of *B. intermedia* from hospital wastewater samples in western Ghana. A hybrid genome assembly was constructed integrating short-read data from DNA Nanoball sequencing with long-read sequences generated by Oxford Nanopore MinION technology. Identification and antimicrobial susceptibility profiles were determined using MicroScan autoSCAN-4 based on Clinical and Laboratory Standard Institute documents. ResFinder and CARD Resistance Gene Identifier (RGI) were used to identify antimicrobial resistance (AMR) genes, and BLAST and VFDB datasets were used to identify virulence factor genes. The complete genome had two chromosomes, no plasmid, and a high average nucleotide identity value (98.05%) with *B. intermedia*. Resistance to trimethoprim-sulfamethoxazole was revealed, the first report in this species. CARD RGI revealed the presence of AMR genes, including ANT(9)-Ic and *adeF*. Local BLAST analysis revealed Cgs, a *B. melitensis* virulence factor. *B. intermedia* is an opportunistic human pathogen clinically isolated several times, suggesting the importance of accurately identifying multidrug resistance. *B. intermedia* may possess virulence factors similar to those of *B. melitensis*. Further study is needed to fully elucidate its pathogenesis.

## 1. Introduction

*Brucella intermedia* (formerly *Ochrobactrum intermedium*) is a gram-negative, aerobic rod found in environmental sources such as soil and water, but it can sometimes be pathogenic to humans. In 2020, all *Ochrobactrum* species were reclassified into the *Brucella* genus, including the species we analyzed in this study [1] and classified as brucellosis-causing *Brucella* species (BBS) and non-brucellosis-causing *Brucella* species (NBBS). Unlike BBS, which are known to cause zoonotic infections, NBBS are primarily found in the environment, such as soil and water, but have been linked to several opportunistic infections. Several virulence factors involved in lipid A biosynthesis, fatty acid biosynthesis, carbohydrate metabolism, cell wall synthesis, and biofilm formation have been identified in *Ochrobactrum* genomes [2], suggesting the significance of analyzing the virulence of NBBS. Moreover, *B. intermedia* retains more virulence-related genes than other species in the genus *Ochrobactrum* [3].

Treatment regimens differ. The first-line antimicrobials for BBS are doxycycline combined with gentamicin; for NBBS, imipenem, fluoroquinolones, trimethoprim-sulfamethoxazole, and aminoglycosides are used [4]. Antimicrobial-resistant BBS are rare, though some resistance has been reported in NBBS [5]. *B. intermedia* is usually resistant to tobramycin, polymyxin B, and colistin [4]. However, data on resistance to other antimicrobials, especially those used for treatment, are lacking.

With increasing antimicrobial use, particularly in developing countries, the risk of antimicrobial resistance (AMR) is growing, with serious consequences, including higher morbidity and mortality. In recent years, environmental AMR has become an important risk factor for public health. AMR spreads to local communities through the microbiome in aquatic environments, and hospital wastewater is a valuable source for monitoring AMR dynamics [6]. Moreover, AMR has become a national problem in Ghana, but the actual situation remains unclear, especially regarding environmental AMR.

In this study, assuming that NBBS isolated from Ghanaian hospital wastewater contribute to the spread of AMR in the region, we conducted a genomic analysis to investigate the virulence and AMR profiles of *B. intermedia*.

## 2. Materials and Methods

### 2.1. Bacterial Source and Identification

Hospital wastewater was collected from one hospital, named Effia Nkwanta Regional Hospital, in western Ghana in 2021. The sample was incubated overnight at 37 °C in 2 mL of tryptic soy broth (TSB) to promote bacterial enrichment. Subsequently, aliquots were plated onto bromothymol blue agar supplemented with colistin (2 µg/mL) and incubated at 37 °C for bacterial isolation. Bacterial isolates were taxonomically identified via 16S rRNA gene sequencing, following the protocol previously described [7]. Briefly, PCR amplification was performed in 10 µL reaction volumes containing 5 µL of 2× Emerald premix (Takara Bio Inc., Shiga, Japan), 0.5 µL each of forward (16S-8UA: AGAGTTTGATCMTGGCTCAG) and reverse (16S-1485B: ACGGGCGGTGTGTRC) primers [7], 3 µL of nuclease-free water, and 1 µL of genomic DNA template. The PCR cycle included an initial denaturation at 98 °C for 1 min, followed by 30 cycles of 98 °C for 5 s, 57 °C for 10 s, and 72 °C for 1 min, and a final extension at 72 °C for 3 min. PCR products were purified using EXOSAP IT (Applied Biosystems, Thermo Fisher Scientific, Tokyo, Japan) and sequenced on a 3730xl DNA Analyzer (Thermo Fisher Scientific, Tokyo, Japan) using the BigDye Terminator v3.1 Cycle Sequencing Kit (Thermo Fisher Scientific, Tokyo, Japan). Resulting sequences were analyzed via BLAST searches against 16S ribosomal RNA sequences (Bacteria and Archaea) of rRNA/ITS databases to determine taxonomic identity. Of the hospital wastewater isolates including bacteria belonging to the families *Enterobacteriaceae* and *Aeromonadaceae*, we analyzed the *B. intermedia* isolate COL9b thereafter.

### 2.2. DNA Extraction and Whole-Genome Sequencing

Genomic DNA was extracted using DNeasy Blood and Tissue Kits and Genomic-tips 20/G (Qiagen, Tokyo, Japan) according to the manufacturer’s protocol. For short-read sequencing, the DNA library was prepared using the MGIEasy FS DNA Library Prep Set (MGI Tech., Beijing, China), loaded onto a DNBSEQ-G400 High-throughput Sequencing Set FCL flowcell, and sequenced on a DNBSEQ-G400RS (MGI Tech.). In total, 12,713,641 reads were obtained. For long-read sequencing, the library was prepared using the Native Barcoding Kit 24 V14 SQK-NBD 114.24 (Oxford Nanopore Technologies, Oxford, UK) following the manufacturer’s protocol. Sequencing was performed on an R10.4.1 flowcell using a MinION Mk1B (Oxford Nanopore Technologies). MinKNOW (version 24.02.8) and Guppy (version 7.3.11; Oxford Nanopore Technologies) were used for base calling and adapter trimming of raw data. Sequences determined by DNBSEQ-G400RS and MinION were assembled using Unicycler v0.5.0 [8]. The complete genomes were deposited in GenBank under Bioproject PRJNA473419, with accession numbers CP183035 and CP183036.

### 2.3. Identification and Antimicrobial Susceptibility Testing

Identification and antimicrobial susceptibility testing were conducted using the MicroScan autoSCAN-4 (Beckman Coulter, Brea, CA, USA) with the prompt method. The antimicrobial susceptibility results were interpreted based on the Clinical and Laboratory Standard Institute (CLSI) documents M100-S24 [9] for colistin and the others for M100-Ed32 [10] for non-Enterobacterales.

### 2.4. Phylogenetic Analysis

Average nucleotide identity (ANI) was calculated using FastANI [11]. Genomic data for 37 *B. intermedia* strains were retrieved from the National Center for Biotechnology Information (NCBI) datasets in June 2024, except for genomes considered isolated from the same samples. A maximum-likelihood phylogenetic tree was constructed using kSNP4.1 [12] and visualized using iTOL v7.

### 2.5. AMR Genes and Virulence Factor Analysis

The genome was annotated using DFAST v1.6.0 [13]. AMR genes were identified using ResFinder [14] and the CARD Resistance Gene Identifier (RGI) [15]. Virulence factors were identified using BLAST v2.15.0 [16] with the VFDB, a comprehensive online resource of literature-based information on bacterial virulence factors, DNA sequences of the core dataset, downloaded on 6 September 2024 [17].

### 2.6. Structure Prediction of Cgs

The amino acid sequence of cyclic ß-1,2 glucan synthase (Cgs) from *Brucella melitensis* was obtained from NCBI (WP_011005413.1). The GT-84 domain sequence was extracted, and its structure was predicted using AlphaFold3 [18]. ChimeraX-1.8 [19] was used to visualize and compare its structure with *B. intermedia*.

## 3. Results

### 3.1. Identification of a Colistin-Resistant Strain from Hospital Wastewater

The *B. intermedia* isolate COL9b was identified using 16S rRNA sequencing, and a BLAST search matched it most closely to *Ochrobactrum intermedium*. Whole-genome sequencing revealed a genome size of 4.8 Mb, consisting of two chromosomes (2.8 Mb and 2.0 Mb), with no plasmid, and a GC content of 57.7%. The genome was annotated with 4684 coding sequences, 12 rRNAs, and 58 tRNAs. ANI analysis showed a high similarity to *B. intermedia* (98.05%).

### 3.2. Phylogenetic Characteristics

**Figure 1:**
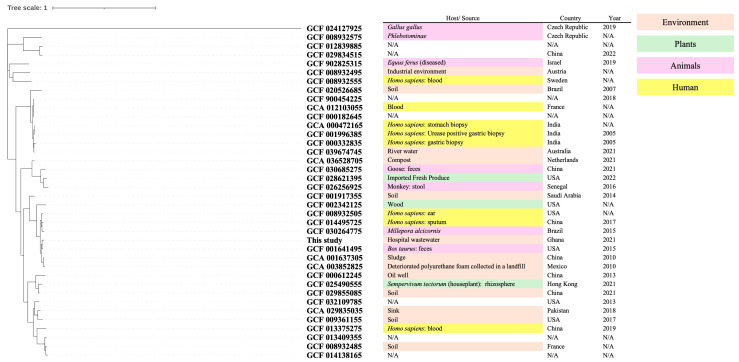
Maximum-likelihood phylogenetic tree of *B. intermedia* genomes. Datasets were obtained from the NCBI database. N/A, Not Available.

The results of the phylogenetic analysis are shown in Figure 1. A phylogenetic tree was constructed using the maximum-likelihood method with the SNP-based analysis tool kSNP4.1, based on genome sequences of the same bacterial species from the NCBI database. The hosts, sources, countries, and years of the isolates are also displayed in Figure 1. As shown on the left-hand side of the table, various environmental and animal sources have been reported for this species, suggesting that the isolate in this study is phylogenetically close to human-derived isolates.

### 3.3. Antimicrobial Susceptibility Testing and AMR Gene Analysis

The antimicrobial susceptibility of COL9b showed resistance to trimethoprim-sulfamethoxazole among NBBS treatment agents (Table 1). *B. intermedia* has an AmpC-type ß-lactamase-producing gene on its chromosome, conferring resistance to penicillin and third-generation cephalosporins, but has been reported to be susceptible to carbapenems, quinolones, aminoglycosides, ciprofloxacin, and trimethoprim-sulfamethoxazole [20]. This is the first report of trimethoprim-sulfamethoxazole resistance in clinical isolates of *B. intermedia*.

There were no hits when ResFinder was used to identify AMR genes. However, the CARD RGI analysis showed four hits (Table 2). Although no genes or mutations directly linked to sulfamethoxazole and trimethoprim resistance were found, genes associated with antibiotic efflux pumps, such as *adeF* (resistance-nodulation-cell division) and *qacG* (small multidrug resistance), were identified.

### 3.4. Virulence Factor Analysis and Putative Function

**Figure 2:**
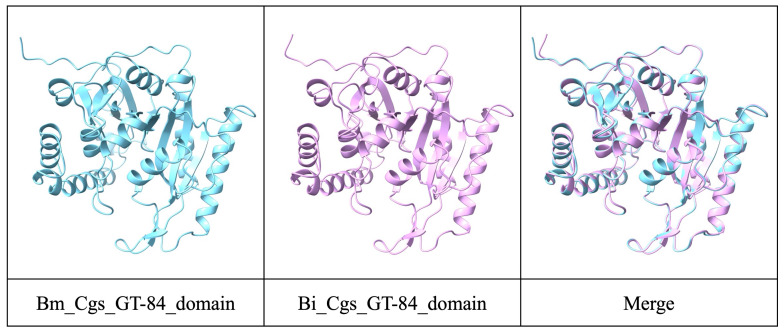
Structure analyses on functional domains of Cgs using AlphaFold3. Analysis was performed with default options.

For virulence factors, we conducted a BLAST search using the VFDB, yielding 4675 hits. We focused on Cgs, a key virulence factor in *B. melitensis*, the primary cause of human brucellosis [21]. The amino acid sequence identity of Cgs in *B. intermedia* isolated in this study was higher than that of *B. abortus* (92.10%) or *B. suis* (92.10%), which cause brucellosis. Because *B. melitensis* is classified as BSL-3, the GT-84 domain, which functions as a Cgs glycotransferase to cyclize glucans [22], was analyzed. Structural predictions for *B. melitensis* (aa 475–818) and *B. intermedia* (aa 492–835) were made using AlphaFold3 (Figure 2). The interface-predicted template modeling (ipTM) scores, representing the accuracy of the predicted relative positions of the subunits within the complex, were 0.90 and 0.91, indicating high-quality predictions. The two models shared nearly identical structures (Figure 2), suggesting these proteins might have the same functions.

## 4. Discussion

The previous name, *O. intermedium*, means the species is between the *Brucella* genus and *Ochrobactrum anthropi*. *B. intermedia* is phylogenetically close to BBS [23]. In this study, we reported the *B. intermedia* isolate COL9b, resistant to trimethoprim-sulfamethoxazole and harboring the putative virulence factor Cgs.

Using MicroScan autoSCAN-4, the isolate was identified as *Ochrobactrum anthropi* (*Brucella anthropi*) with a probability of 93.99%; however, in the ANI analysis, it was recognized as *Brucella intermedia* as previously described. Since *B. anthropi* and *B. intermedia* differ in their susceptibility to certain antimicrobials, *B. intermedia* is resistant to tobramycin, polymyxin B, and colistin [4]. Therefore, accurate identification is required for the treatment.

The following five main mechanisms mediate bacterial resistance to trimethoprim-sulfamethoxazole: (1) the permeability barriers and/or efflux pumps, (2) naturally insensitive target enzymes, (3) regulatory changes in the target enzymes, (4) mutational or recombinational changes in the target enzymes, and (5) acquired resistance by antimicrobial-resistant target enzymes [24]. *adeF* and *qacG* are multidrug efflux components, so they are assumed to be related to trimethoprim-sulfamethoxazole resistance of this isolate. Moreover, ANT(9)-Ic inactivates some aminoglycosides, which may contribute to intermediate susceptibility to amikacin. Recently, AAC-(6′)-laq was identified in *B. intermedia* isolate in China [25], but we did not identify it, suggesting that *B. intermedia* has a variety of AMR genes depending on the regions or sources. Notably, this is the first documented case of a trimethoprim-sulfamethoxazole-resistant *B. intermedia* isolate, which affected the selection of antimicrobial agents for treatment.

Cyclic ß-1,2 Glucan (CßG) is one of the virulence factors of *Brucella* spp. for successful host infection. The CßG synthesis pathway comprises three proteins: Cgs, cyclic glucan transporter (Cgt), and cyclic glucan modifier (Cgm) [26]. We demonstrated that *B. intermedia* may possess a protein with a function similar to that of *B. melitensis* Cgs; however, Cgt and Cgm are required for successful intracellular invasion. NBBS are free-living bacteria [27], and some non-classical intracellular *Brucella* spp. have been isolated from animals and humans [28]. Annotation results suggested that the isolate possesses proteins with high homology to Cgm and Cgt; therefore, the *B. intermedia* isolate in this study has the potential to invade host cells with CßG.

Hospital wastewater is recognized as a reservoir of AMR genes [29] and contributes to their dissemination to local communities. Trimethoprim-sulfamethoxazole remains in various wastewaters, such as wastewater treatment plant influents and livestock farming wastewater effluents [30]. It is possible that the *B. intermedia* isolate in this study was resistant to trimethoprim-sulfamethoxazole in this environment, as it contains various antimicrobials and other substances that can lead to an environmental response. In addition, the dissemination of AMR in developing countries is rooted in people’s inappropriate behavior toward antimicrobials, including limited laboratory infrastructure, a lack of appropriate functioning drug regulatory mechanisms, and non-human use of antimicrobials [31]. We need to monitor AMR attitudes carefully, not only within hospitals but also throughout the environment in developing countries.

## 5. Conclusions

In conclusion, *B. intermedia* isolated in this study was resistant to trimethoprim-sulfamethoxazole and a multidrug-resistant strain bearing AMR genes such as ANT(9)-Ic and *adeF*. Moreover, we suggested that *B. intermedia* has a virulence factor that might function in the same manner as Cgs of *B. melitensis*. Phylogenetically related strains to this isolate have been disseminating globally across human, animal, and environmental reservoirs; therefore, it is necessary to accumulate data on *B. intermedia*, and continuous monitoring is essential from a One Health perspective.

## Figures and Tables

**Table 1:** Antimicrobial susceptibility. The breakpoints were based on the CLSI documents M100-Ed32 and M100-S24. S, susceptible; I, intermediate; R, resistant.

Antimicrobials	MIC (μg/mL)	Interpretation
Piperacillin	>64	R
Piperacillin-tazobactam	>64	R
Ceftazidime	>16	R
Cefepime	>16	R
Aztreonam	>16	R
Imipenem	≤2	S
Meropenem	≤2	S
Gentamicin	4	S
Tobramycin	>8	R
Amikacin	32	I
Minocycline	≤2	S
Ciprofloxacin	0.5	S
Levofloxacin	≤0.5	S
Trimethoprim-sulfamethoxazole	>2/38	R
Colistin	>4	R

**Table 2:** Antimicrobial resistance genes identified by CARD RGI. Data type was DNA sequence, and the criterion was “Perfect and Strict hits only”.

ARO Term	AMR Gene Family	Drug Class	Resistance Mechanism	% Identity of Matching Region	% Length of Reference Sequence
*adeF*	resistance-nodulation-cell division (RND) antibiotic efflux pump	fluoroquinolone antibiotic, tetracycline antibiotic	antibiotic efflux	48.12	99.24
ANT(9)-Ic	ANT(9)	aminoglycoside antibiotic	antibiotic inactivation	97.68	100
*qacG*	small multidrug resistance (SMR) antibiotic efflux pump	disinfecting agents and antiseptics	antibiotic efflux	43.27	102.8
*adeF*	resistance-nodulation-cell division (RND) antibiotic efflux pump	fluoroquinolone antibiotic, tetracycline antibiotic	antibiotic efflux	71.29	100.19

## Data Availability

The datasets presented in this study can be found in online repositories at the National Center for Biotechnology Information (NCBI) BioProject database under accession number PRJNA473419. The accession numbers for the BioSamples of SAMN47009341 are CP183035 and CP183036.

## Abbreviations

The following abbreviations are used in this manuscript:

AMR	Antimicrobial Resistance
ANI	Average Nucleotide Identity
BBS	Brucellosis-Causing *Brucella* Species
BLAST	Basic Local Alignment Search Tool
Cgs	Cyclic ß-1,2 Glucan Synthase
CLSI	Clinical and Laboratory Standards Institute
DNBSEQ	DNA Nanoball Sequencing
NBBS	Non-Brucellosis-Causing *Brucella* Species
PCR	Polymerase Chain Reaction
RGI	Resistance Gene Identifier
VFDB	Virulence Factor Database
